# Prevalence of intestinal colonization and nosocomial infection with carbapenem-resistant *Enterobacteriales* in children: a retrospective study

**DOI:** 10.3389/fpubh.2023.1251609

**Published:** 2023-11-23

**Authors:** Fen Pan, Pengcheng Chen, Yuxin Duan, Fangyuan Yu, Wenhao Weng, Hong Zhang

**Affiliations:** ^1^Department of Clinical Laboratory, Shanghai Children's Hospital, School of Medicine, Shanghai Jiao Tong University, Shanghai, China; ^2^Institute of Pediatric Infection, Immunity, and Critical Care Medicine, Shanghai Jiao Tong University School of Medicine, Shanghai, China; ^3^Suzhou Molarray Co., Ltd, Jiangsu, China

**Keywords:** carbapenem-resistant *Enterobacteriales*, intestinal colonization, nosocomial infection, KPC-2, NDM-1

## Abstract

**Objective:**

We investigated the epidemiological surveillance of the intestinal colonization and nosocomial infection of carbapenem-resistant *Enterobacteriales* (CRE) isolates from inpatients, which can provide the basis for developing effective prevention.

**Methods:**

A total of 96 CRE strains were collected from 1,487 fecal samples of hospitalized children between January 2016 and June 2017, which were defined as the “CRE colonization” group. In total, 70 CRE clinical isolates were also randomly selected for the comparison analysis and defined as the “CRE infection” group. The antimicrobial susceptibility of all strains was determined by the microdilution broth method. Polymerase chain reaction (PCR) was used to analyze carbapenemase genes, plasmid typing, and integrons. Multilocus sequence typing was further used to determine clonal relatedness.

**Results:**

In the “CRE colonization” group, *Klebsiella pneumoniae* was mostly detected with a rate of 42.7% (41/96), followed by *Escherichia coli* (34.4%, 33/96) and *Enterobacter cloacae* (15.6%, 15/96). The ST11 KPC-2 producer, ST8 NDM-5 producer, and ST45 NDM-1 producer were commonly present in carbapenem-resistant *K. pneumoniae* (CRKPN), carbapenem-resistant *E. coli* (CRECO), and carbapenem-resistant *E. cloacae* (CRECL) isolates, respectively. In the “CRE infection” group, 70% (49/70) of strains were *K. pneumoniae*, with 21.4% *E. cloacae* (15/70) and 5.7% *E. coli* (4/70). The ST15 OXA-232 producer and ST48 NDM-5 producer were frequently observed in CRKPN isolates, while the majority of NDM-1-producing CRECL isolates were assigned as ST45. Phylogenetic analysis showed that partial CRE isolates from intestinal colonization and nosocomial infection were closely related, especially for ST11 KPC-2-producing CRKPN and ST45 NDM-1-producing CRECL. Furthermore, plasmid typing demonstrated that IncF and IncFIB were the most prevalent plasmids in KPC-2 producers, while IncX3/IncX2 and ColE were widely spread in NDM producer and OXA-232 producer, respectively. Then, class 1 integron intergrase *intI1* was positive in 74.0% (71/96) of the “CRE colonization” group and 52.9% (37/70) of the “CRE infection” group.

**Conclusion:**

This study revealed that CRE strains from intestinal colonization and nosocomial infection showed a partial correlation in the prevalence of CRE, especially for ST11 KPC-2-producing CRKPN and ST45 NDM-1-producing CRECL. Therefore, before admission, long-term active screening of rectal colonization of CRE isolates should be emphasized.

## Background

Enterobacteriales represent a large family of gram-negative bacteria, which cause a series of nosocomial infections including pneumonia, bloodstream infections, and urinary tract infections. Carbapenems including ertapenem, imipenem, and meropenem are commonly considered the last line of defense to treat multidrug-resistant bacterial infections, and the wide and unreasonable use of these antibiotics has consequently resulted in the increasing incidence of antimicrobial resistance. Carbapenem-resistant *Enterobacteriales* (CRE) are regarded as the top-ranked bacteria in the priority list of resistant pathogens of the World Health Organization and have become a severe threat to global public health (1, 2). In China, the overall incidence of CRE infection per 10,000 discharges was 4.0 and differed significantly by region (3). Production of carbapenemases including *Klebsiella pneumoniae* carbapenemase (KPC) and New Delhi metallo-β-lactamase (NDM) was the main mechanism of CRE. Then, the rapid spread of CRE severely limited the options for antibiotics and prolonged the length of hospital stay, ultimately resulting in high mortality.

The family *Enterobacteriales* naturally colonizes in the gastrointestinal tract, which is the most important reservoir for CRE strains. Previous studies reported that the prevalence of CRE colonization rates was less than or ~5%, but it would be higher in critical patients in the intensive care unit (4–7). Furthermore, fecal carriage of CRE isolates is regarded as a risk factor for developing CRE infections. A recent study showed that 50% of the patients with CRE rectal colonization could develop CRE-associated pneumonia and simultaneously were more likely to have a long hospital stay and high hospitalization expense than patients with non-colonized CRE (8). Therefore, early and rapid detection of CRE colonization among high-risk patients will be helpful to implement strategies to prevent CRE dissemination.

Many guidelines recommend that active screening of rectal colonization of CRE is an easy and important tactic to control CRE infection, and rectal swabs are considered as the preferred sample for screening on account of good patient compliance and fewer side effects (9, 10). Accordingly, we started the CRE screening for hospitalized children in 2016 and continuously monitored the change in CRE prevalence. However, the distribution of characteristics between the colonized CRE and clinical CRE isolates among children still needs to be summarized. In this study, we will investigate epidemiological surveillance of the colonization/infection of CRE isolates from inpatients, which can provide the basis for developing effective prevention measures for CRE infections.

## Methods

### Study design and bacterial isolates

To compare the colonization/infection of CRE isolates from inpatients, this study was designed in Shanghai Children's Hospital between January 2016 and June 2017. The inclusion criteria were as follows: Fecal sample was collected from hospitalized children aged 0 to 18 years who received the routine fecal culture testing, and only the first fecal sample of each inpatient was enrolled. The exclusion criteria were as follows: (1) patients older than 18 years; (2) patients from an outpatient clinic; and (3) patients with duplicate fecal samples. A total of 1,487 fecal samples from 1,487 inpatients were transported to clinical microbiology for fecal culture testing and were further screened by performing the meropenem disk screening method. Moreover, 96 CRE strains were detected with positive results, which were defined as the “CRE colonization” group. Meanwhile, we randomly selected 70 CRE clinical isolates from the 210 clinical CRE isolates using the random number generation function in Microsoft Office Excel 2010 (Microsoft Corporation, Redmond, WA, USA), and these CRE strains were isolated from different samples including sputum, blood, urine, and pus, which caused nosocomial infections. These clinical isolates were incorporated for the comparison analysis and defined as the “CRE infection” group. The clinical information of all enrolled inpatients including patient demographics, hospital ward, use of antibiotics, and treatment outcomes was also collected from electronic medical records. All CRE isolates were identified by the matrix-assisted laser desorption/ionization time-of-flight mass spectrometry (MALDI-TOF-MS) system using MALDI Biotyper (Bruker Daltonik GmbH, Bremen, Germany). These isolates were stored at −80°C in a 40% glycerol broth medium for further analysis.

### Antimicrobial susceptibility testing

The minimum inhibitory concentrations (MICs) of antimicrobial agents including cefazolin, cefuroxime, ceftazidime, ceftriaxone, cefotaxime, cefepime, ampicillin–sulbactam, piperacillin–tazobactam, ertapenem, imipenem, meropenem, aztreonam, ciprofloxacin, levofloxacin, amikacin, gentamicin, trimethoprim–sulfamethoxazole, ceftazidime–avibactam, tigecycline, and colistin were determined by the microdilution broth method. The results were interpreted by the breakpoints of Clinical and Laboratory Standards Institute (CLSI) 2019. The interpretive criterion for tigecycline was based on the breakpoints of the Food and Drug Administration (FDA). *Escherichia coli* ATCC 25922 and *E. coli* ATCC 35218 were used for quality control.

### Detection of carbapenemase genes

All the CRE isolates were screened for the presence of carbapenemase genes including *bla*_NDM_, *bla*_KPC_, *bla*_OXA − 48_, *bla*_IMP_, *bla*_AIM_, *bla*_VIM_, *bla*_GIM_, and *bla*_SIM_ by polymerase chain reaction (PCR) as previously described (11) (Supplementary Table 1S). The amplicons were sequenced, and nucleotide sequences were further analyzed and compared with the sequences available at the National Center for Biotechnology Information (NCBI) website (https://blast.ncbi.nlm.nih.gov/Blast.cgi).

### Multilocus sequence typing

Genetic evolutionary relationships were identified for *K. pneumoniae, E. coli*, and *Enterobacter cloacae* using multilocus sequence typing (MLST) schemes. The housekeeping genes of *K. pneumoniae* (*ropB, gapA, mdh, pgi, phoE, infB*, and *tonB*) and *E. coli* (*dinB, icdA, pabB, polB, putB, trpA, trpB*, and *uidA*) were performed according to the protocol available on the website of Institute Pasteur MLST (https://bigsdb.pasteur.fr/). Meanwhile, amplification of housekeeping genes for *E. cloacae* was also conducted as described on the PubMLST website (https://pubmlst.org/). The STs were assigned by sequencing and submitting the sequences to the MLST database for alignment. MEGA 7.0 software was used for Phylogenetic analysis.

### Plasmid replicon analysis

For all CRE isolates, the molecular typing of the plasmids was performed using the PCR-based replicon typing, and 17 pairs of primers (HI1, HI2, I1, L/M, N, FIA, FIB, W, Y, P, FIC, A/C, T, FIIAs, F, K, and B/O) were amplified as described previously. In addition, we used new primers typing IncX groups to IncX1-IncX4 and new primers for ColE-type plasmid (12–14). The amplified products were electrophoresed in 1.5% agarose gels, stained with Green Nucleic Acid Stain (Sangon, Shanghai, China), and visualized under ultraviolet light using the Gel Doc 2000 system (Bio-Rad).

### Detection and sequencing of integrons

The class 1, 2, and 3 integrons were screened by PCR amplification for *intI1, intI2*, and *intI3* genes. Furthermore, amplification of the *intI1* variable region was performed using the primers 5′-CS and 3′-CS under the conditions and annealing temperatures as described previously (15, 16) (Supplementary Table 1S). To identify different types of gene cassettes, same-sized amplicons were analyzed by restriction fragment length polymorphism (RFLP) with digested *Rsa*I and *Hinf* I genes (TaKaRa Bio Inc., Tokyo, Japan), which were dependent on the species. Amplicons showing the same pattern were considered to be identical gene cassettes, and then, one representative product of each distinct RFLP was purified and sequenced by Sangon Biotech Co. Ltd (Shanghai, China). The sequencing results were analyzed using the BLAST program on the NCBI website (https://blast.ncbi.nlm.nih.gov/Blast.cgi).

### Statistical analysis

The statistical analysis was performed using SPSS 25.0 for Windows (version 25.0; SPSS Inc., Chicago, IL, USA). For continuous variables, data were described as median, which was compared using the Mann–Whitney *U*-test. Multivariate analysis was used to identify the independent predictors, and we calculated the odds ratios (ORs) and the 95% confidence intervals (CIs) for each variable. A value of *P* ≤ 0.05 was considered statistically significant.

## Results

### Clinical characteristics of enrolled patients

A total of 96 non-duplicate samples were detected as CRE-positive with a carriage of 6.5% (96 out of 1,487). The clinical characteristics of enrolled patients between the “CRE colonization” group and the “CRE infection” group are presented in Table 1. The CRE strains between the two groups were predominantly isolated from the department of neonatology, with severe underlying diseases including pneumonia, sepsis, and gastrointestinal infections. More than 50% of patients in the “CRE colonization” group and the “CRE infection” group had a history of invasive procedures and antibiotic exposures such as carbapenems (*P* ≤ 0.05). It is worth noting that 14.6% of CRE carriers in the “CRE colonization” group had CRE infections after the detection of CRE. The death rates between the “CRE colonization” group and the “CRE infection” group were 2.1 and 2.9%, respectively. Multivariate analysis showed that the underlying diseases of gastrointestinal infections (OR = 0.044, 95% CI: 0.006–0.342, *P* = 0.003) and invasive procedures (OR = 180.081, 95% CI: 16.681–1,944.066, *P* < 0.001; Table 2) are independent risk factors for the colonization/infection of CRE isolates.

**Table 1:** Clinical characteristics of patients with CRE positive in this study.

**Clinical characteristics**	**CRE colonization group (*n* = 96)**	**CRE infection group (*n* = 70)**	*P*-value^*^
Male sex	56 (58.3%)	38 (54.3%)	0.603
Age in months (median)	12 m	1 m	0.010
**Isolation wards**			
Neonatology dept.	33 (34.4%)	45 (64.3%)	<0.001
General medicine	18 (18.8%)	6 (8.6%)	0.066
PICU	17 (17.7%)	11 (15.7%)	0.735
Hematology department	16 (16.7%)	3 (4.3%)	0.013
Respiratory department	6 (6.3%)	1 (1.4%)	0.127
Others	6 (6.3%)	4 (5.7%)	0.886
**Underlying diseases**			
Pneumonia	58 (60.4%)	48 (68.6%)	0.280
Sepsis	23 (24.0%)	25 (35.7%)	0.099
Gastrointestinal infections	37 (38.5%)	7 (10.0%)	<0.001
UTI	5 (5.2%)	6 (8.6%)	0.390
Cephalomeningitis	4 (4.2%)	7 (10.0%)	0.136
Hospitalization stays (median)	11 days	37 days	0.079
Recent hospitalization history	39 (40.6%)	21 (30.0%)	0.159
Invasive procedures	48 (50.0%)	59 (84.2%)	<0.001
**Antibiotic exposures**			
β-lactam/β-lactamase inhibitor	44 (45.8%)	41 (58.6%)	0.105
Cephalosporins	63 (65.6%)	36 (51.4%)	0.066
Carbapenems	35 (36.5%)	39 (55.7%)	0.014
CRE infection	14 (14.6%)	–	–
**Clinical outcome**			
Improved	63 (65.6%)	66 (94.3%)	<0.001
Unhealed	31 (32.3%)	2 (2.9%)	0.000
Died	2 (2.1%)	2 (2.9%)	0.748

* A comparison between two groups. A P-value of ue. A on between inhibitorstically significant.

CRE, Carbapenem-resistant Enterobacteriales, PICU, pediatric intensive care unit; UTI, urinary tract infection.

**Table 2:** Multivariate analysis for factors associated with CRE.

**Variables**	**OR**	**95% CI**	*P*-value^*^
Underlying diseases (gastrointestinal infections)	0.044	0.006–0.342	0.003
Invasive procedures	180.081	16.681–1,944.066	<0.001

* A P-value of ue.05 was considered statistically significant.

OR, odds ratio; CI, confidence interval.

### Distribution of CRE isolates from intestinal colonization and nosocomial infection

**Figure 1:**
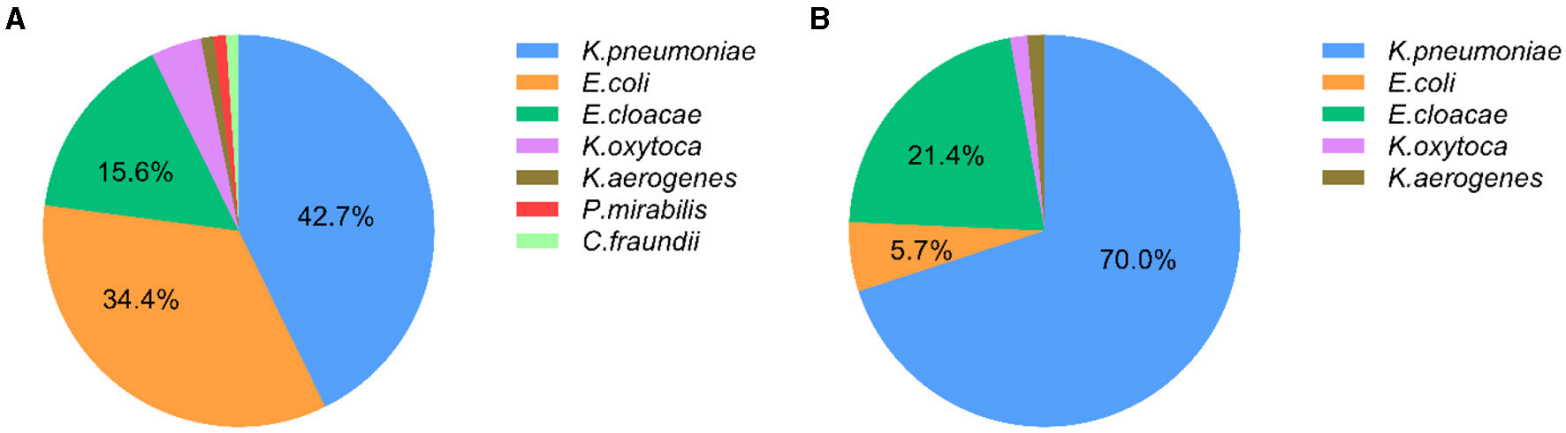
Distribution of carbapenem-resistant *Enterobacteriales* (CRE) in inpatients with CRE intestinal colonization and CRE infection. **(A)** The distribution of CRE strains from intestinal colonization; **(B)** The distribution of CRE strains from nosocomial infection.

Among 96 isolates enrolled in the “CRE colonization” group, *K. pneumoniae* was mostly detected with a rate of 42.7% (41/96), followed by *E. coli* (34.4%, 33/96) and *E. cloacae* (15.6%, 15/96). Other species including four *Klebsiella oxytoca*, one *Klebsiella aerogenes*, one *Proteus mirabilis*, and one *Citrobacter freundii* were also observed in the intestinal tract of children, as shown in Figure 1A. Meanwhile, 70% (49/70) strains in the “CRE infection” group were *K. pneumoniae*, with 21.4% *E. cloacae* (15/70), 5.7% *E. coli* (4/70), 1.4% *K. oxytoca* (1/70), and 1.4% *K. aerogenes* (1/70; Figure 1B). *K. pneumoniae* is commonly isolated not only from children with CRE intestinal colonization but also from children with CRE nosocomial infection.

### Carbapenemase and antimicrobial susceptibility of isolates

**Figure 2:**
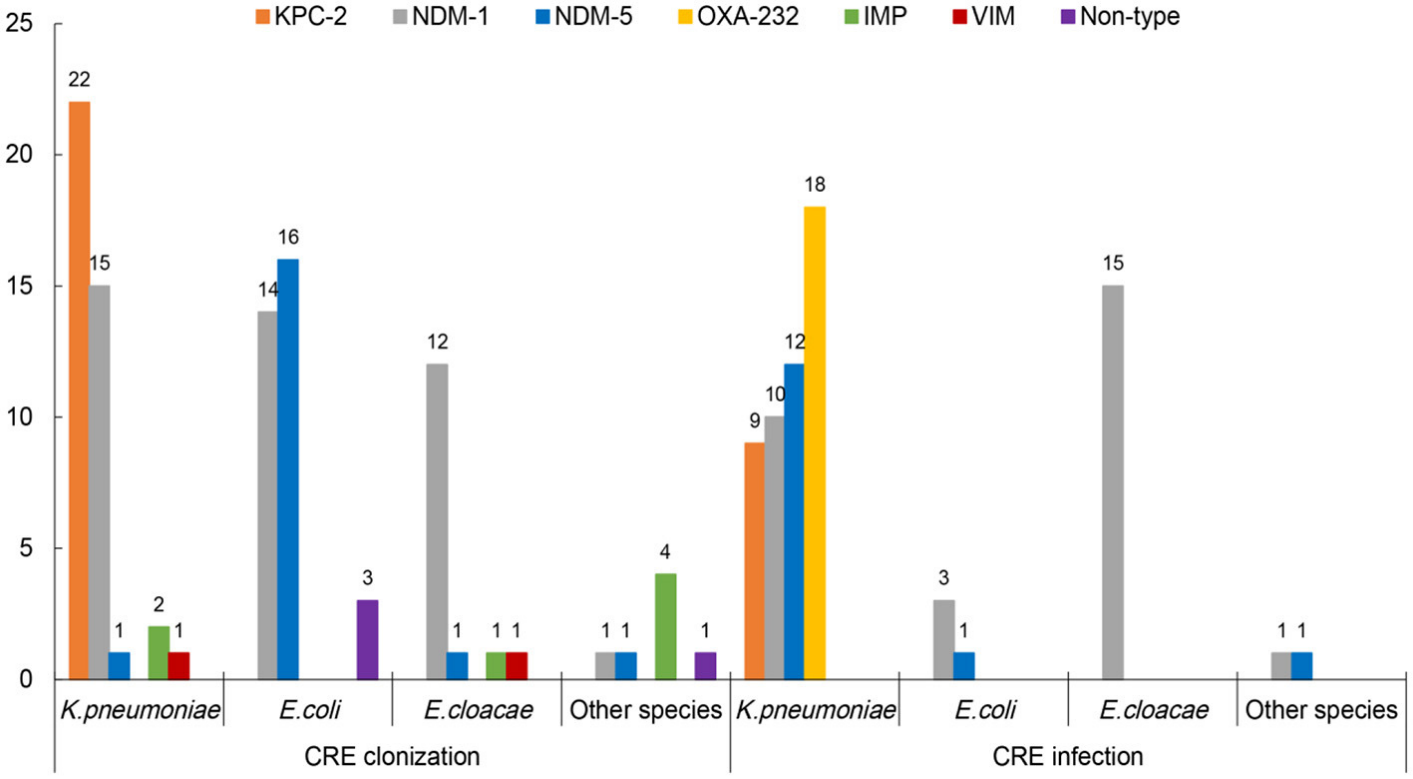
The carbapenemases of carbapenem-resistant *Enterobacteriales* (CRE) strains from intestinal colonization and nosocomial infection.

In the “CRE colonization” group, 95.8% (92/96) of CRE isolates produced carbapenemases. NDM-1 was the major carbapenemase (43.8%), with KPC-2, NDM-5, and IMP being 22.9%, 19.8%, and 7.3%, respectively. KPC-2 and NDM-1 were observed in 53.7% (22/41) and 36.6% (15/41) of carbapenem-resistant *K. pneumoniae* (CRKPN) isolates, while NDM-5 and NDM-1 account for 48.5% (16/33) and 42.4% (13/33) of carbapenem-resistant *E. coli* (CRECO) strains, respectively. The NDM-1 was most frequently found in carbapenem-resistant *E. cloacae* (CRECL; Figure 2). However, in the “CRE infection” group, NDM-1, OXA-232, NDM-5, and KPC-2 account for 41.4%, 25.7%, 20%, and 12.9% of CRE isolates, respectively. The majority of OXA-232, NDM-5, NDM-1, and KPC-2 were detected in CRKPN strains, with four CRECO strains and fifteen CRECL strains containing NDM-1 carbapenemase (Figure 2).

**Figure 3:**
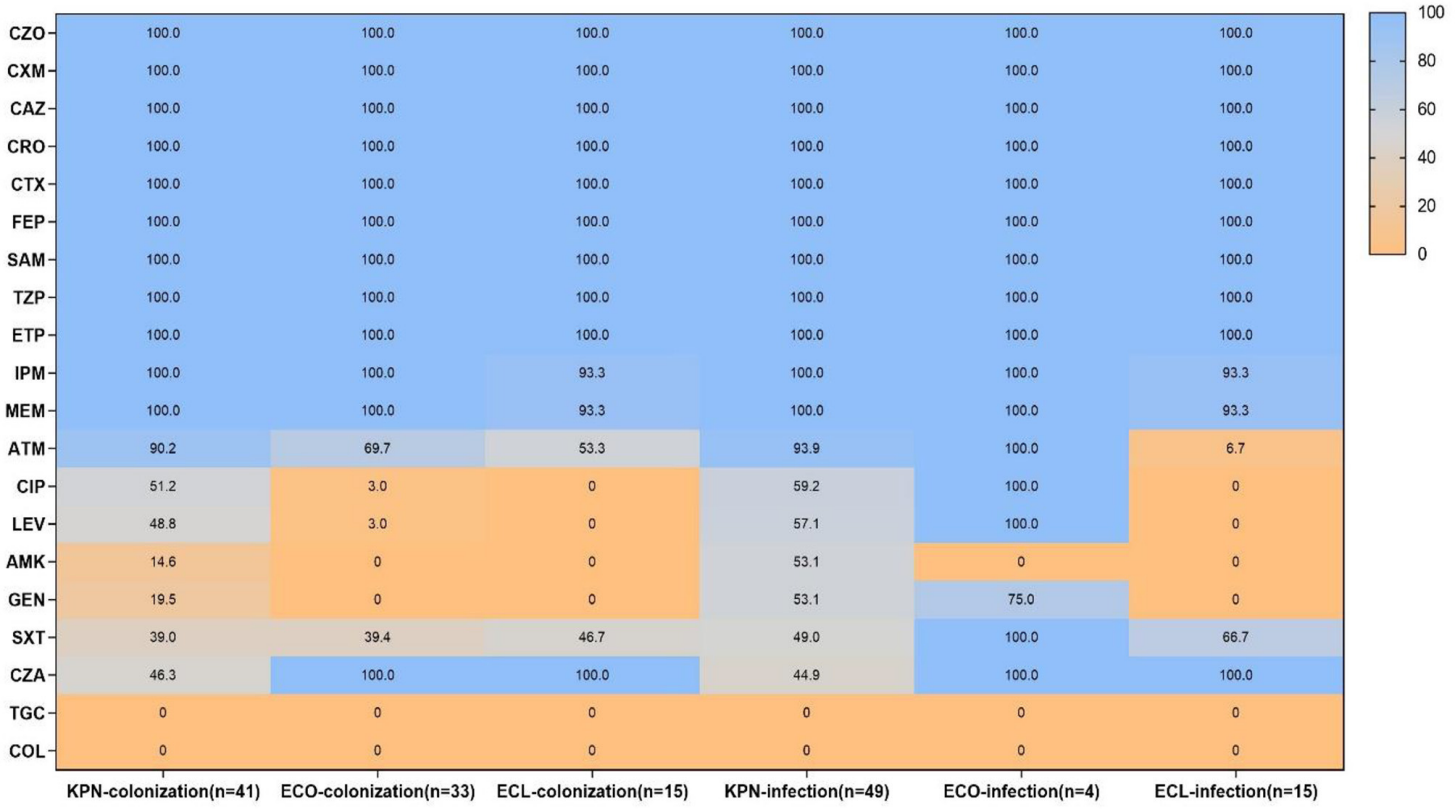
Antimicrobial resistance pattern of carbapenem-resistant *Enterobacteriales* (CRE) strains from intestinal colonization and nosocomial infection. KPN, *Klebsiella pneumoniae*; ECO, *Escherichia coli*; ECL, *Enterobacter cloacae*; CZO, cefazolin; CXM, cefuroxime; CAZ, ceftazidime; CRO, ceftriaxone; CTX, cefotaxime; FEP, cefepime; SAM, ampicillin-sulbactam; TZP, piperacillin–tazobactam; ETP, ertapenem; IPM, imipenem; MEM, meropenem; ATM, aztreonam; CIP, ciprofloxacin; LEV, levofloxacin; AMK, amikacin; GEN, gentamicin; SXT, trimethoprim-sulfamethoxazole; CZA, ceftazidime-avibactam; TGC, tigecycline; COL, colistin.

**Figure 4:**
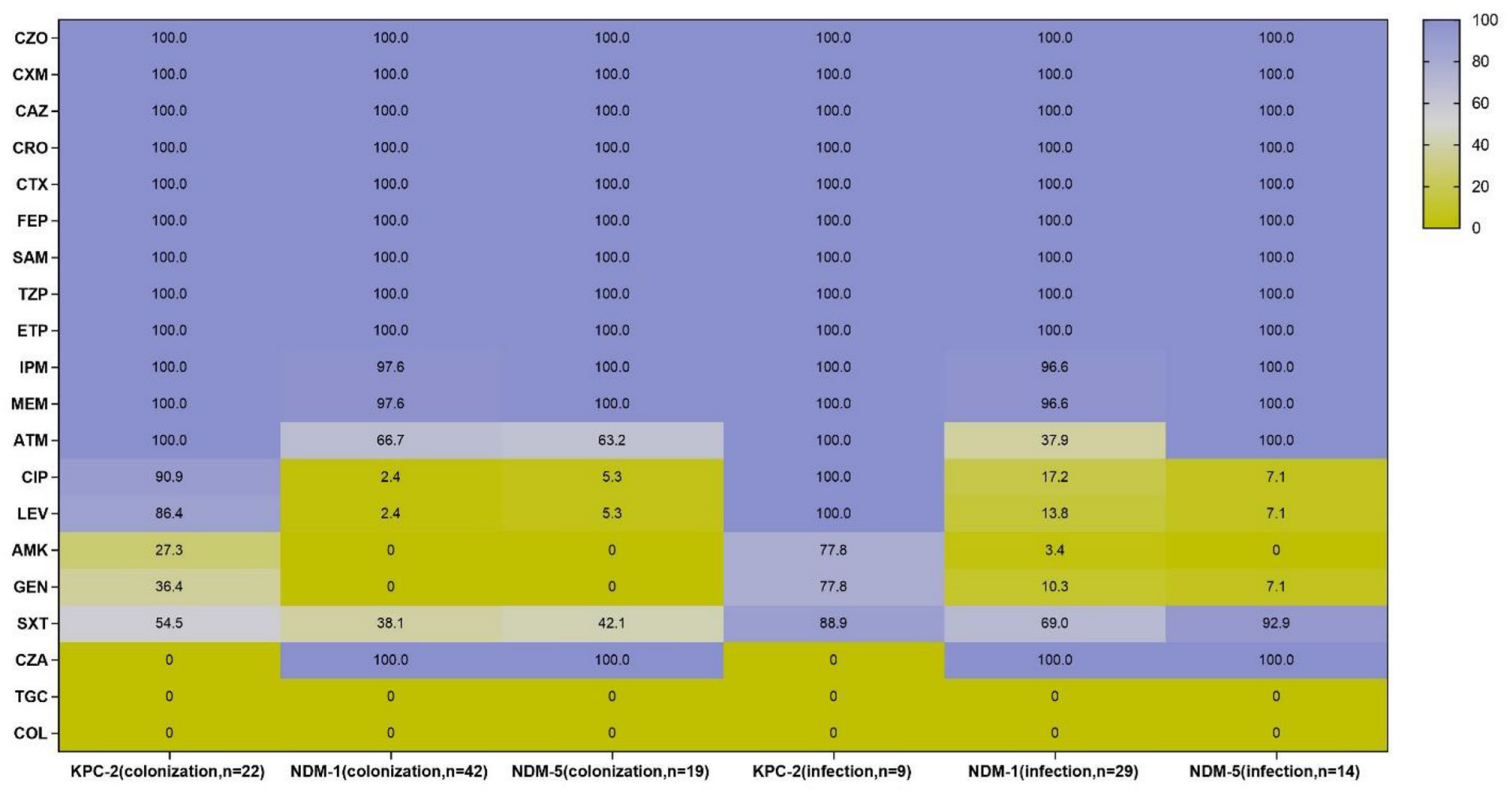
Antimicrobial resistance profiles of KPC-2, NDM-1, and NDM-5-producing strains from intestinal colonization and nosocomial infection. CZO, cefazolin, CXM, cefuroxime, CAZ, ceftazidime, CRO, ceftriaxone, CTX, cefotaxime, FEP, cefepime, SAM, ampicillin-sulbactam, TZP, piperacillin–tazobactam, ETP, ertapenem, IPM, imipenem, MEM, meropenem, ATM, aztreonam, CIP, ciprofloxacin, LEV, levofloxacin, AMK, amikacin, GEN, gentamicin, SXT, trimethoprim-sulfamethoxazole, CZA, ceftazidime-avibactam, TGC, tigecycline; COL, colistin.

Antimicrobial susceptibility results demonstrated that all CRE isolates exhibited high resistance to cephalosporins and carbapenems, as well as ampicillin–sulbactam and piperacillin–tazobactam, while all strains were susceptible to tigecycline and colistin. However, the resistant pattern of different species from intestinal colonization and nosocomial infection varied, with CRKPN isolates being the most serious. As shown in Figure 3, CRKPN isolates in the “CRE infection” group showed higher resistance to amikacin and gentamicin than these species showed in the “CRE colonization” group. The *in vitro* activity of aztreonam, ciprofloxacin, levofloxacin, and trimethoprim–sulfamethoxazole against CRECO isolates in the “CRE infection” group was higher than these antimicrobial strains in the “CRE colonization” group. Of note, the resistance spectrum of KPC-2-producing isolates was different from the other type of carbapenemase, especially for ciprofloxacin, levofloxacin, amikacin, gentamicin, and trimethoprim–sulfamethoxazole (Figure 4).

### Molecular phenotype and plasmid replicons of isolates

**Figure 5:**
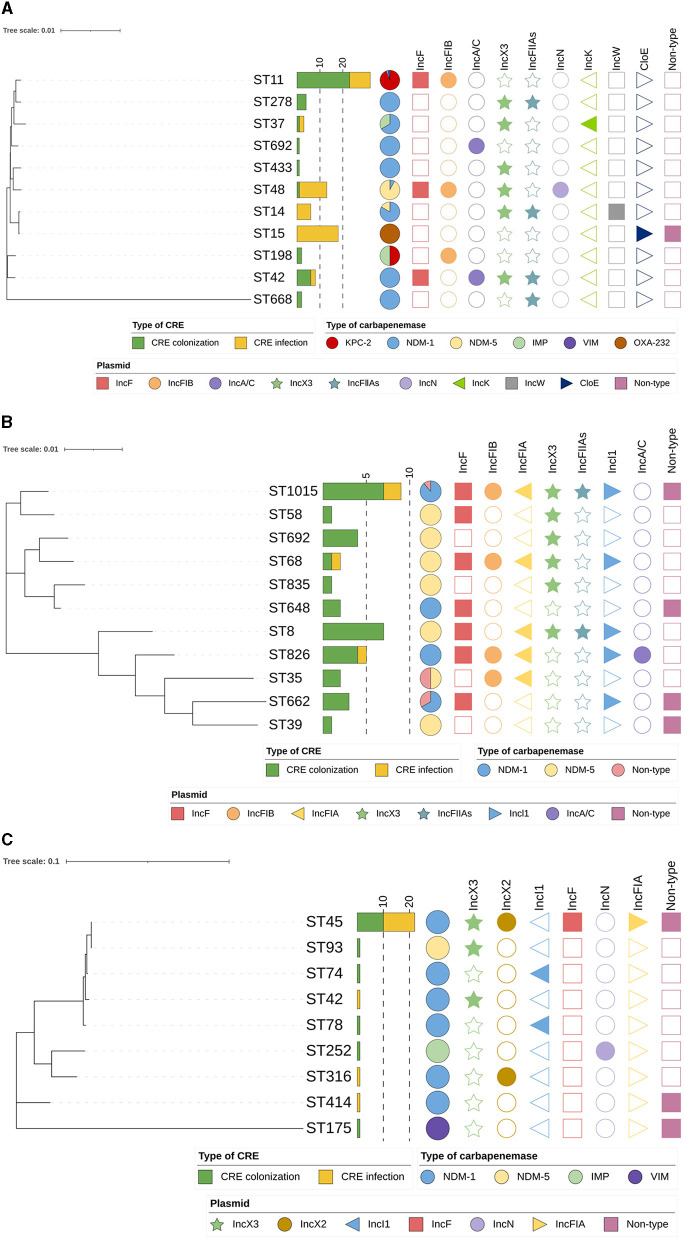
Phylogenetic tree of MLST data among CRE isolates analyzed by MEGA 7. **(A)** Phylogenetic tree of carbapenem-resistant *Klebsiella pneumoniae*; **(B)** Phylogenetic tree of carbapenem-resistant *Escherichia coli*; **(C)** Phylogenetic tree of carbapenem-resistant *Enterobacter cloacae*.

According to the MLST analysis, CRE isolates were assigned to various ST types in different species (*K. pneumoniae*, 11 STs; *E. coli*, 11 STs; and *E. cloacae*, nine STs; Figure 5). Noticeably, ST11 (95.4%, 21/22) was the most predominant type in KPC-2-producing *K. pneumoniae* in the “CRE colonization” group, while ST15 and ST48 were frequently represented ST in OXA-232-producing *K. pneumoniae* and NDM-5-producing *K. pneumoniae* isolates in the “CRE infection” group, respectively. Moreover, the distribution of STs was relatively dispersive in carbapenemase-producing *E. coli* between the two groups, with 41.2% (7/17) of NDM-5 producers and 47.1% (8/17) of NDM-1 producers being typed as ST8 and ST1015, respectively. Additionally, the majority of CRECL strains harboring NDM-1 carbapenemase were assigned to ST45 in “CRE colonization” (83.3%, 10/12) and “CRE infection” groups (73.3%, 11/15). Phylogenetic analysis showed that partial CRE isolates from intestinal colonization and nosocomial infection were closely related, especially for ST11 KPC-2-producing CRKPN and ST45 NDM-1-producing CRECL.

The plasmid replicon types of CRE isolates showed that isolates containing particular carbapenemase carried different types of plasmids (Figure 5). Of CRKPN isolates, most ST11 strains (81.3%, 26 out of 32) had plasmid replicon-type IncF together with IncFIB, which harbored the *bla*_KPC − 2_ gene. ST48 isolates (92.3%, 12 out of 13) and ST15 isolates (94.4%, 17 out of 18) carried IncX3 plasmid and ColE plasmid, respectively. Among CRECO isolates, IncX3 and IncA/C plasmids were relatively highly observed in several ST types. However, 54.5% of ST45 *bla*_NDM − 1_ carrying CRECL isolates possessed plasmid replicon-type IncX2.

### Characteristics of integrons

In the “CRE colonization” group, class 1 integron intergrase *intI1* was detected in 71 isolates (74.0%, 71 out of 96), with 6 different gene cassettes. The prevalence of complex class 1 integrons varied in different species. The most common gene cassette in CRKPN and CRECL isolates was *dfrA*12-*orf*-*aadA*2, while *aar*3-*dfrA*27 was the most detected gene among CRECO isolates. However, in the “CRE infection” group, 37 isolates (52.9%, 37/70) were observed positive with class 1 integron, and the gene cassettes *dfrA*12-*orf*-*aadA*2 and *aadA*1-*aacA*6 were common in CRKPN and CRECL isolates, respectively. No isolate was detected with class 2 integron intergrase *intI2* and class 3 integron intergrase *intI3*.

## Discussion

Recently, the CRE isolates have been increasingly identified in children, and their horizontal spread among different species has contributed to a dilemma of no drugs available for clinical anti-infective therapy. Children are considered a special population, and once they are infected with CRE strains, it will greatly limit the choice of antimicrobial agents. Colonization of CRE strains in the gastrointestinal tract implies the potential for nosocomial transmission, and rapid active screening of CRE is essential for reducing and controlling CRE infections. Notably, the proportion of CRE colonization in hospitalized patients ranged from 15.5 to 52% and varied among different countries, different crowds, and different departments (17–20). This study revealed a carriage rate of 6.5%, which was lower than the previous literature. Furthermore, evaluating the special risk factors for the colonization/infection of CRE isolates is crucial for identifying high-risk patients. Consistent with other studies (21, 22), invasive procedures such as mechanical ventilation were identified as risk factors for colonization or infection with CRE. Additionally, long hospitalization stays and exposure to antibiotic use also increase the likelihood of cross-transmission of CRE isolates.

The diversity of CRE bacterial species was observed in this study, and the most common CRE isolates both in intestinal colonization and nosocomial infection was CRKPN, which is in agreement with the findings of previous studies (23, 24). Notably, the rapid increase of CRKPN in recent years is a critical threat to global health and causes high mortality (25). In addition, the proportion of CRECO in the “CRE colonization” group was higher than those in the “CRE infection” group, while the proportion of CRECL in the “CRE colonization” group was lower than those in the “CRE infection” group. It could be speculated that CRECO isolates were easier to detect in fecal samples, but it should be further verified by expanding the number of specimens. CRECL represents the third most common CRE followed by CREKPN and CRECL isolates, and the increased incidence of these isolates has constantly been reported worldwide, which requires more clinical attention (26, 27).

The prevalence of carbapenemase types among CRE strains has varied by regions: metallo-β-lactamases (MBLs) including NDM and VIM were dominant in isolates collected in Africa, the Middle East, and Asia/Pacific; KPC was mainly detected in Latin America and North America; and OXA-48-like carbapenemases were predominant in Europe (23). However, a study conducted in China displayed that the most prevalent carbapenemase gene was *bla*_KPC − 2_ among *K. pneumoniae* isolates from adult patients and *bla*_NDM_ among *E. coli* isolates from children (28). In this study, among CRKPN isolates, KPC-2 producers were the most detected in the “CRE colonization” group, while OXA-232 producers were predominantly found in the “CRE infection” group. Notably, KPC-2-bearing ST11 CRKPN isolates were widely disseminated throughout China (28, 29) and had been reported to be colonized in the intestinal tract (30). In recent years, OXA-48-like carbapenemase, including OXA-162 and OXA-181, was increasingly prevalent in CRE strains (31, 32), and OXA-232 carbapenemase, a variant of OXA-181 with one amino acid substitution, was found in ST15 *K. pneumoniae* strains in Shanghai, China (33). Even though in this study, the OXA-48-like carbapenemase was not distributed in colonized CRKPN isolates, the clinicians should still be aware of the emergence of OXA-48-like carbapenemase in the gastrointestinal tract, which was predominantly distributed in fecal colonization (34). Interestingly, the frequency of NDM-carrying isolates has increased worldwide, and NDM-type has spread globally and is detected in various species. Among CRECO strains, NDM-type carbapenemase, including NDM-1 and NDM-5, was frequently detected in the “CRE colonization” group and the “CRE infection” group. Meanwhile, NDM-1-type carbapenemase was the most common among ST45 CRECL isolates both in the “CRE colonization” group and the “CRE infection” group, showing the high similarity of CRECL between colonization and infection. The trend of carbapenemase types in the colonization/infection of CRE isolates should be further concerned.

Mobile genetic elements containing integrons and plasmids highlight their role in the horizontal spread of carbapenemase genes among CRE isolates (35). Other resistant genes in mobile genetic elements also contribute to resistance to antibiotics and result in therapeutic failure of antibiotics. This study revealed that various plasmids were detected in CRE isolates, and no difference in the distribution of plasmids was found between the “CRE colonization” group and the “CRE infection” group. A high incidence of IncF-type plasmid in *bla*_KPC − 2_-carrying CRKPN isolates was observed, while IncX type, especially IncX3 and IncX2, was observed in most *bla*_NDM_-carrying CRE isolates. The presence of IncF and IncX3 plasmid-mediated the clonal dissemination of carbapenemase genes reported throughout the world (36, 37). Furthermore, integrons with classes 1–3 were known as multidrug-resistant integrons that may ultimately contribute to the wide spread of “superbugs” including CRE (38). A high frequency of class 1 integrons was identified in the “CRE colonization” group (74.0%) and the “CRE infection” group (52.9%), which is in agreement with an earlier report (16). Urgent surveillance of mobile genetic elements among CRE isolates should be conducted to control the spread of these pathogens.

Our study still has several limitations. First, this study was performed in one hospital, and the results were not suitable for other institutions. Therefore, multicenter design and comprehensive capture of the colonization/infection of CRE isolates should be further conducted. Second, even though we recommended CRE screening for all hospitalization patients, the active screening was still not frequently performed for all inpatients. The fecal sample was collected from hospitalized children who received the routine fecal culture testing. It might have led to the low CRE-positive rate. Finally, the sample size was relatively small and could not fully reflect the prevalence of intestinal colonization and nosocomial infection of CRE strains.

## Conclusion

In conclusion, this study described the prevalence of intestinal colonization and nosocomial infection with CRE isolates in children, and KPC-2, NDM-1, NDM-5, and OXA-232 carbapenemases were the main types found in the “CRE colonization” group and the “CRE infection” group. Then, a few intestinal colonized CRE strains were probably linked to clinically infected strains, especially for ST11 KPC-2-producing CRKPN and ST45 NDM-1-producing CRECL. Therefore, in the case of the outbreak of these high-risk CRE clones from intestinal colonization, we should reinforce the surveillance of CRE from intestinal colonization and long-term active screening of rectal colonization of CRE isolates before admission should also be emphasized.

## Data availability statement

The raw data supporting the conclusions of this article will be made available by the authors, without undue reservation.

## Ethics statement

This study was approved by the Ethics Committee of Shanghai Children's Hospital (Shanghai, China) [code: 2019R031-F01].

## Author contributions

FP, WW, and HZ designed this study. FP, PC, YD, and FY conducted methodology, acquisition of data, and data analysis. FP wrote the first draft of the manuscript. WW and HZ critically revised and edited the manuscript. All authors read and approved the final manuscript.
