# Supplementary material for: Prevalence of intestinal colonization and nosocomial infection with carbapenem-resistant Enterobacteriales in children: a retrospective study
Source: Front Public Health. 2023 Nov 23;11:1251609. doi: 10.3389/fpubh.2023.1251609 (PMC10702246; doi:10.3389/fpubh.2023.1251609)
Supplement: Supplementary file 1 [file Table_1.doc]

Table 1S. Primers of carbapenemase genes and integron genes

| Primer | Primers(5’→3’) | Target gene(s) or region |
| --- | --- | --- |
| *bla*_NDM_-F | ATGGAATTGCCCAATATTATGC | *bla*_NDM_ |
| *bla*_NDM_-R | TCAGCGCAGCTTGTCGGC |
| *bla*_KPC_-F | AGGACTTTGGCGGCTCCAT | *bla*_KPC_ |
| *bla*_KPC_-R | TCCCTCGAGCGCGAGTCTA |
| *bla*_OXA-48_-F | GCGTGGTTAAGGATGAACAC | *bla*_OXA-48_ |
| *bla*_OXA-48_-R | CATCAAGTTCAACCCAACCG |
| *bla*_IMP_-F | GGAATAGAGTGGCTTAAYTCTC | *bla*_IMP_ |
| *bla*_IMP_-R | GGTTTAAYAAAACAACCACC |
| *bla*_VIM_-F | GATGGTGTTTGGTCGCATA | *bla*_VIM_ |
| *bla*_VIM_-R | CGAATGCGCAGCACCAG |
| *bla*_AIM_-F | CTGAAGGTGTACGGAAACAC | *bla*_AIM_ |
| *bla*_AIM_-R | GTTCGGCCACCTCGAATTG |
| *bla*_GIM_-F | TCGACACACCTTGGTCTGAA | *bla*_GIM_ |
| *bla*_GIM_-R | AACTTCCAACTTTGCCATGC |
| *bla*_SIM_-F | TACAAGGGATTCGGCATCG | *bla*_SIM_ |
| *bla*_SIM_-R | TAATGGCCTGTTCCCATGTG |
| *intl1*-F | GCATCCTCGGTTTTCTGG | *i**ntI1* |
| *intl1*-R | GGTGTGGCGGGCTTCGTG |  |
| 5’-CS | GGCATCCAAGCAGCAAG | Class 1 integron variable region |
| 3’-CS | AAGCAGACTTGACCTGA |
| *intl2*-F | CACGGATATGCGACAAAAAGGT | *intI2* |
| *intl2*-R | GTAGCAAACGAGTGACGAAATG |
| *intl3*-F | ATCTGCCAAACCTGACTG | *intI3* |
| *intl3*-R | CGA ATGCCCCAACAACTC |
